# Synthesis and Characterization of Hyperbranched and Organosilicone Modified Waterborne Polyurethane Acrylates Photosensitive Resin

**DOI:** 10.3390/polym13132039

**Published:** 2021-06-22

**Authors:** Na Wang, Xinhui Wang, Jinyan Lang, Zhenhua Hu, Heng Zhang

**Affiliations:** 1 College of Marine Science and Biological Engineering, Qingdao University of Science & Technology, Qingdao 266042, China; wlalala21@163.com (N.W.); wxhameq@163.com (X.W.); ljy17806248212@163.com (J.L.); 2 School of Food Engineering, Ludong University, Yantai 264025, China; zhenhuahu1991@163.com; 3 Key Laboratory of Biomass Chemical Engineering of Ministry of Education, Zhejiang University, Hangzhou 310027, China

**Keywords:** hyperbranched, organosilicone modified, waterborne, polyurethane acrylates, photosensitive resin

## Abstract

A new type of waterborne polyurethane acrylate was synthesized for use as a UV curing coating. The N,N-dihydroxy methyl ethyl-3-Methyl aminopropanoate monomer was first prepared via adding reactions of methyl acrylate and diethanol amine with methyl alcohol as the solvent. Then, the hyperbranched prepolymer was obtained by addition of trimethylolpropane with toluenesulfonic acid as catalyst and N,N-dimethyl formamide as solvent. The resulting hyperbranched and organosilicone modified waterborne polyurethane acrylates was synthesized through the mixed reaction of prepolymer and Hydroxy silicone oil, polyethylene glycol-1000, toluene diisocynate, dimethylolpropionic acid, 1,2-propylene glycol, hydroxyethyl acrylate, and triethylamine with dibutyltin dilaurate as the catalyst. The molecular structures were characterized by FT-IR and ^1^H NMR spectroscopy and GPC analysis and the thermal stability was studied by using TGA. Moreover, the influence of contemodnt of hydroxyl silicone oil, dimethylolpropionic acid, polyethylene glycol-1000, and prepolymer to various of properties such as glossiness, hardness, adhesive force, abrasion resistance, water absorption, elongation at break and tensile strength of films were analyzed. The temperature and catalyst dosage impact on percent conversion of isocyanate group (–NCO) were also studied. It was proven that the best dosage of hydroxyl silicone oil and dimethylolpropionic acid were 4.6%, the dosage of polyethylene glycol-1000 was 50%, and the amount of hyperbranched prepolymer was 0.5%, which could make the film achieve the optimum properties. The percent conversion of isocyanate group (–NCO) was maximum when reacting two hours at 80 °C with 0.2% catalyst.

## 1. Introduction

UV curing technology is widely used in chemical industry, electronics, aerospace, communication, machinery, and other fields due to its advantages of fast curing speed, simple equipment, automatic operation, and less environmental pollution [1,2,3]. Among the materials applied in UV curing technology, the polyurethane acrylate [4] (PUA) is a type of waterborne polyurethane and acrylate copolymer emulsion with excellent comprehensive performance and core-shell structure [5] and it is also a kind of photosensitive resin [6] which is widely used and studied nowadays. PUA has the high adhesion and wear resistance of polyurethane resin [7], and the water resistance, corrosion resistance, and good flexibility of acrylic resin [8,9,10]. This is especially true of the environmentally friendly waterborne polyurethane acrylate produced after water-based urethane acrylate (WPUA), which is safe and has better mechanical properties and compatibility. However, the waterborne PUA leads to poor water resistance and poor mechanical and optical properties, which will hinder the popularization and application of WPUA in various fields. Hence, an improvement of WPUA is of great significance.

Part of the synthesis of hyperbranched polyurethane acrylate has been studied, and it has the advantages of low viscosity, high solubility, fast light curing, and great thermal stability. For instance, Johansson and Hult [11] synthesized a series of hyperbranched polyurethane acrylates. This kind of polymer was found to have suitable melt rheology for low temperature powder coatings, which makes it well applied in UV curing coatings. Qi [12] used the hyperbranched polyester made in the laboratory to modify the polyurethane acrylate and obtained the hyperbranched modified polyurethane acrylate with low viscosity and good mechanical properties such as tensile strength, elongation at break, and hardness. He [13] found that compared to unmodified resins, hyperbranched polyurethane modified acrylic resins greatly improve the overall performance and the speed of UV curing, and the hydrophilic properties of the resin are proportional to the content of hydrophilic groups. Asif [14] synthesized a series of novel hyperbranched waterborne polyurethane acrylates with good thermal stability and low viscosity via adding some hydroxyl groups of hyperbranched polyester to the acid groups of acrylates. Qiuxue [15] added acrylate to the hyperbranched polyester polyol modified polyurethane to obtain hyperbranched waterborne polyurethane acrylate with good water resistance, dynamic mechanical properties, and thermal stability. With the advancement of research on hyperbranched polyurethane acrylate, its performance has gradually improved, but there are few studies on waterborne polyurethane acrylate with excellent comprehensive performance. In this study, waterborne polyurethane acrylate with excellent comprehensive properties has been obtained through modification, and its application field has been expanded.

Organosilicon compounds have hydrophobicity, low surface tension, and a stable structure, which can be used in waterproof coatings to effectively prevent water penetration. Furthermore, silicon also has great thermal stability, biocompatibility, biological oxidation stability, and excellent mechanical and processing properties [16,17,18,19,20]. Javaherian [21] et al. synthesized a series of WPUA containing silicon that can improve the thermal stability and found that the water resistance of WPUA increased with the increase of silicon content. Guangmei [22] prepared the polyurethane modified by acrylate/nano-SiO_2_ composites by in-situ polymerization and sol-gel process, and the mechanical properties and thermal stability of it were improved obviously. Photosensitive silicone polyurethane acrylate was synthesized by Sun [23] et al. through infrared method. Also, the effect of the photoinitiator and active monomer on the photopolymerization properties of photosensitive silicone polyurethane acrylate system was studied. Nevertheless, these synthetic processes need to a lot of diluent to reduce the viscosity of the polymer, which not only reduces the performance of cured film, causes shrinkage, cracking and other problems, but also leads to environmental pollution.

Compared with novel waterborne UV-curable hyperbranched polyurethane acrylate/silica [24], the hyperbranched and organosilicone modified waterborne polyurethane acrylates photosensitive resin in this study is through copolymerization to introduce Si-O-Si bond into polyurethane, and it has the advantages of excellent thermal stability and good gloss.

Unfortunately, above research results cannot get waterborne PUA with excellent comprehensive performance yet. In this paper, a new type of hyperbranched silicon modified waterborne PUA was synthesized by combining the fine performance of hyperbranched PUA and silicone modification. It also has the advantages of environmental protection, photosensitivity, high strength, and low viscosity, which can improve the performance of PUA, make up for its defects, and provide a new way for the modification of PUA.

## 2. Experimental

### 2.1. Materials

Methanol, p-toluenesulfonic acid, polyethylene glycol-1000 (PEG-1000), and di-n-butylamine were purchased from Sinopharm Chemical Reagent Co., Ltd. Shanghai, China N,N-dimethylformamide, methyl acrylate, toluene, isopropanol, 1,2-propanediol and benzophenone were purchased from Tianjin Bodi Chemical Co., Ltd., Tianjin, China Trimethylolpropane was obtained from Jining Baichuan Chemical Co., Ltd., Shandong, China. Diethanolamine was obtained from Shanghai Minchen Chemical Co., Ltd., Shanghai, China Acetone, triethylamine, dibutyltin dilaurate (DBTDL), hydrochloric acid, anhydrous sodium carbonate, and ethanol (95%) were purchased from Laiyang Economic and Technological Development Zone Fine Chemical Plant. Toluene; ldiisocyanate (TDI) was purchased from Yantai Juli Isocyanate Co., Ltd., Shandong, China. Hydroxy silicone oil was purchased from Shandong Dayi Chemical Co., Ltd., Shandong, China. β-Hydroxyethyl Acrylate (HEA) was obtained from Qingzhou Better Chemical Group Co., Ltd., Shandong, China. Tripropylene glycol diacrylate was purchased from Taiwan Changxing Chemical Co., Ltd., Kaohsiung City, Taiwan. The above reagents are of analytical grade.

The % we use below is the percentage by weight which is the ratio of the mass of the substance in question to its total mass.

### 2.2. Synthesis of Hydroxyl-Terminated Hyperbranched Polymer

(1) First, 45.6 g (0.53 mol) methyl acrylate and 52.5 g (0.50 mol) diethanolamine were added to perform Michael addition reaction, and methanol was added as solvent. In this reaction, methyl acrylate should be slightly excessive to ensure complete reaction of diethanolamine. Under the condition that with the protection of N_2_, the raw materials were first stirred at room temperature at low speed for about 1 h, then the temperature was raised to 45 °C, reacted for 4.5 h, and the methanol and excess methyl acrylate were removed by rotary evaporation. Then, a colorless and transparent methyl N,N-dihydroxyethyl-3-aminopropionate monomer was obtained.
(2) 57.3 g (0.3 mol) N,N-dihydroxyethyl-3-aminopropionic acid methyl ester monomer and 13.4 g (0.1 mol) trimethylolpropane (TMP) were added to synthesize hyperbranched polymers with trimethylolpropane as the cores. 0.4 g p-toluenesulfonic acid was added as catalyst, 15 g N,N-dimethylformamide was used as solvent, and the raw material was stirred and heated to 110 °C for 3.5 h. The methanol generated during the reaction was removed with a water trap to ensure the progress of the reaction. The solvent N,N-dimethylformamide was removed by rotary evaporation to obtain a pale yellow hydroxyl-terminated hyperbranched polymer.

### 2.3. Synthesis of Hyperbranched Silicon Modified Waterborne Polyurethane Acrylate

**Figure 1:**
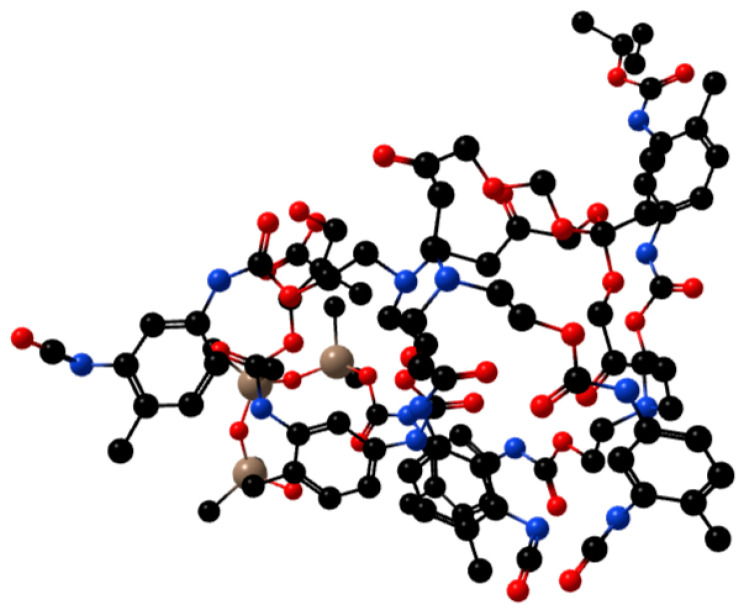
3D molecular structure formula of WPUA.

A certain amount of hydroxyl-terminated hyperbranched polymer, hydroxy silicone oil, polyethylene glycol-1000 (PEG-1000), toluene diisocyanate (TDI), dimethylolpropionic acid (DMPA), 1,2-propanediol, and dibutyl tin dilaurate (DBTDL) as the catalyst were added to the four-mouth burner equipped with a constant pressure funnel, a thermometer, a spherical condenser, and a stirrer. Under the protection of nitrogen, the temperature was raised to a certain temperature. After a period of reaction, β-hydroxyethyl acrylate (HEA) was added. During the reaction, triethylamine was added, and the reaction reached the end to obtain a light-yellow liquid product. The molecular structure of the product is shown in Figure 1.

### 2.4. Preparation of Coating Film

The hyperbranched silicon-modified waterborne polyurethane acrylate oligomer was dissolved in the reactive diluent tripropylene glycol dipropylene (TPGDA), and a certain amount of benzophenone was added as photoinitiator. The SZQ-2 type four-blade wet film preparation machine was used to coat the glass plate (the film thickness is 100 μm) and the ultraviolet light curing was carried out on the UV curing machine with the wavelength of 365 nm and the light intensity of 4 W/cm^2^ to obtain the ultraviolet light curing film.

### 2.5. Determination and Characterization

After the sample was dissolved in acetone, the infrared spectroscopy was measured by a 510P FT-IR infrared spectrometer. Deuterated acetone was added to dissolve the sample, and DRX600 nuclear magnetic resonance spectrometer was used for nuclear magnetic resonance spectrometry. A proper amount of the sample was taken, and the temperature was increased at a constant rate of 10 °C/min within the temperature range of 0 °C to 650 °C, and the thermal weight loss was determined by the TG209F1 thermogravimetric analyzer. Polystyrene as the standard substance and tetrahydrofuran as the mobile phase, the standard sample and the sample to be tested were configured into a solution that was 3–5 mg/mL. Under the condition of passing N_2_, the sample was injected at a constant speed, and the gel chromatography was carried out with a 515–2410 gel chromatograph. The change of -NCO content [25] was measured during the reaction. Then, the performance of the photocurable film was tested: the glossiness of the coated paper surface was detected with gloss tester under the measuring angle of 75°; the hardness [26] of light curing film was tested by the hardness of pencil; adhesion was measured with a hexameter according to ISO standards; abrasion resistance was tested by grinding wheel, and the opaque coating was qualified after testing; the water absorption rate was determined by compared the weight change of the sample after weighing water absorption; and the tensile strength and elongation at break [27] of the film were measured on a tensile tester.

## 3. Results and Discussion

### 3.1. Infrared Characterization of Water-Borne Polyurethane Acrylate Modified by Hyperbranched Silicon

**Figure 2:**
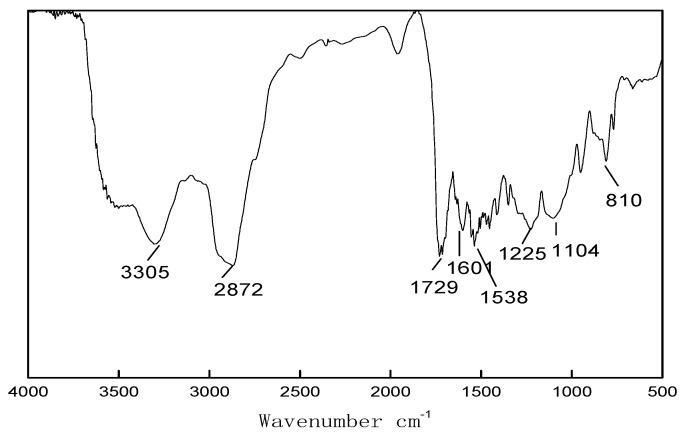
FT-IR spectra of water-borne polyurethane acrylate modified by hyperbranched silicon.

As can be seen from Figure 2, the absorption peak of C-SI group was found at 810 cm^−1^ site, the stretching vibration absorption peak at 1104 cm^−1^, the C=C absorption peak which is weak at 1601 cm^−1^, the characteristic absorption of the amide group at 3305 cm^−1^ and 1454–1538 cm^−1^, the stretching vibration absorption peak of C-H at 2872 cm^−1^, -COO absorption peak near 1729 cm^−1^, and the absorption peak of NCO should be near 2250 cm^−1^, but it does not show up on the IR spectrum, which means that NCO has been added completely.

### 3.2. Nuclear Magnetic Characterization of Water-Borne Polyurethane Acrylate Modified by Hyperbranched Silicon

**Figure 3:**
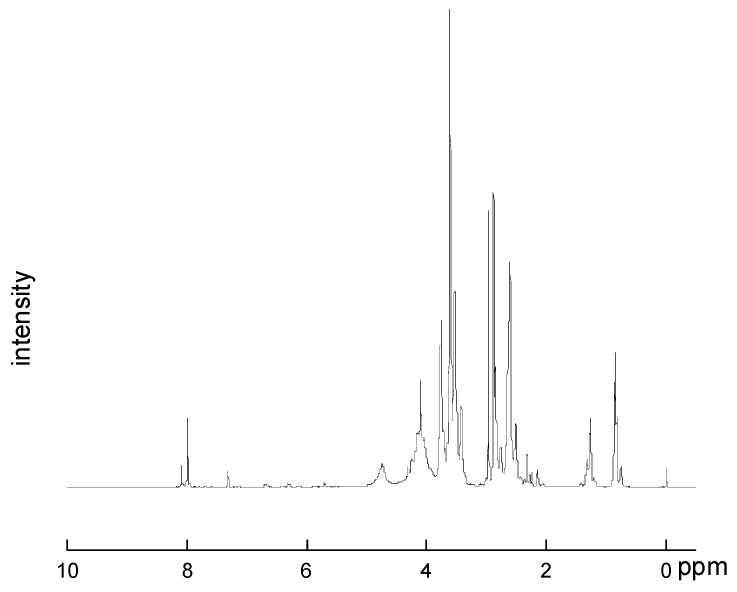
^1^HNMR spectra of water-borne polyurethane acrylate modified by hyperbranched silicon.

As can be seen from Figure 3, ^1^H NMR (δ, CD_3_COCD_3_): 5.785–6.296 ppm, -CH=CH_2_, among them, 6.296–6.259 ppm: H_2_C=C**H**-, 6.040–6.095 ppm: H_a_**H_b_**C=CH-, 5.785–5.805 ppm: **H_a_**H_b_C=CH-, 4.098–4.295 ppm: -C**H_a_**H_b_-, 1.946–2.146 ppm: -CH_a_**H_b_**-, 3.49–3.5 ppm: N-**H**.

According to the figure, an obvious characteristic peak appears at 3.49–3.5 ppm, therefore, the N-H bond that does not exist in the raw material is generated in the reaction, indicating the reaction of -NCO, so the product is determined.

### 3.3. Thermogravimetric Analysis

**Figure 4:**
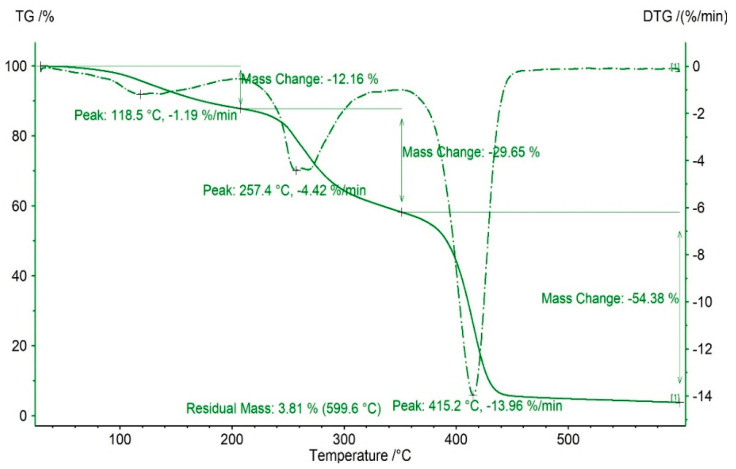
Thermogravimetric (TG) and derivative thermogravimetric (DTG) curve of water-borne polyurethane acrylate modified by hyperbranched silicon.

Figure 4 shows the thermal stability curve of the UV curable film. It can be seen from the TG curve that when the temperature is 0~220 °C, the sample appears at first weightlessness, and the weightlessness rate is 12.16%. In the temperature range of 220~360 °C; with the increase of temperature, the sample appears the second time of weightlessness, which is mainly caused by the chemical bond fracture of the sample molecules, such as N-H bond. At 360~450 °C, the sample appears the third time to lose weight, and the rate of losing weight is large, the rate of losing weight is 54.38%. At 599.6 °C, the sample decomposes completely, and the weight loss rate is 96.19%. From the DTG curves, it can be seen that the change of thermal weight loss rate is the biggest at 118.5 °C, 257.4 °C and 415.2 °C, being 1.19 %/min, 4.42 %/min and 13.96 %/min respectively. At 220 °C, 360 °C, and 450 °C, the change rate of thermal weightlessness is 0.

The first thermogravimetry of the sample is found in the temperature range of 0~220 °C, which is mainly caused by the decomposition of small molecular weight substances in the sample (mainly small molecules of alcohol and β-hydroxyethyl acrylate, etc.), resulting in the weight loss of the sample. In the temperature range of 220~360 °C, with the rise of temperature, the sample appears the second time of weightlessness, mainly due to the N-H bond fracture in the sample molecules. In the temperature range of 360~450 °C, with the increase of temperature, the sample appears the third time of weightlessness, the main reason is that the C=O bond in the molecule and the C=C bond break, resulting in weightlessness. Due to the large number of C=O bond and C=C bond in the molecule, the weight loss rate of the sample in this stage is high. When the temperature reaches 599.6 °C, the sample remains 3.81%, which is basically completely decomposed.

It can be concluded that due to the introduction of hyperbranched polymer and silicone materials, the crosslinking degree in the molecular structure of the oligomer is increased, and the heat resistance of the polyurethane acrylate without hyperbranched and silicon modification (the heat resistance temperature is generally 450 °C [28]) is significantly improved. The oligomer of hyperbranched silicon modified polyurethane acrylate decomposes completely at 599.6 °C, the thermal weight loss rate is 96.19%, and the thermal stability of the oligomer is good.

### 3.4. Gel Chromatographic Characterization

**Figure 5:**
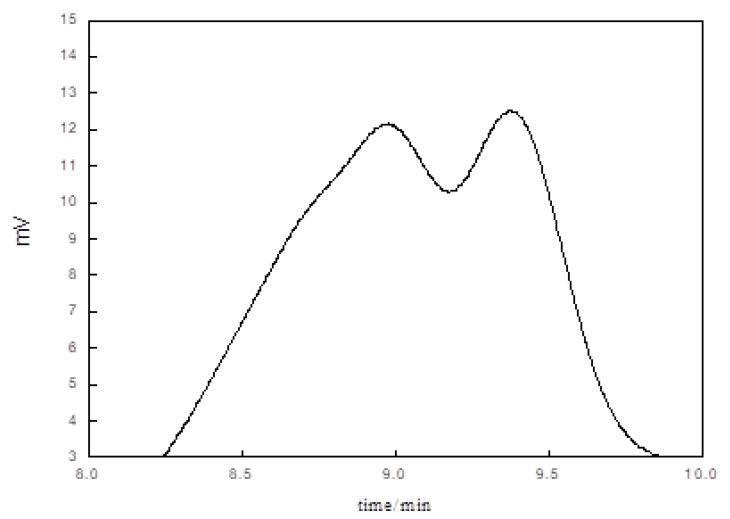
Gel Chromatography of water-borne polyurethane acrylate modified by hyperbranched silicon.

Figure 5 shows the GPC curve of the hyperbranched silicon-modified water-based polyurethane acrylate oligomer (ordinate is the detection signal size, and abscissa is the injection time). The bimodal peak appears in the figure, and the distribution is wide. The reason is that branched cross-linking occurs between monomer molecules, resulting in a wide molecular weight of the product. Because of the three-dimensional structure of hyperbranched polymer, the molecular weight increases. MN (number average molecular weight) is 8631, MW (weight average molecular weight) is 10,561, Mw/Mn (polydispersity index) is 1.224. MW > GT in consequence of the long molecular chain of Oligomer. MW/MN (polydispersity index) is 1.224, and the molecular weight distribution is narrow, which indicates that the chain length and molecular chain length are uniform.

### 3.5. Effect of the Amount of Hydroxy Silicone Oil on Product Properties

**Figure 6:**
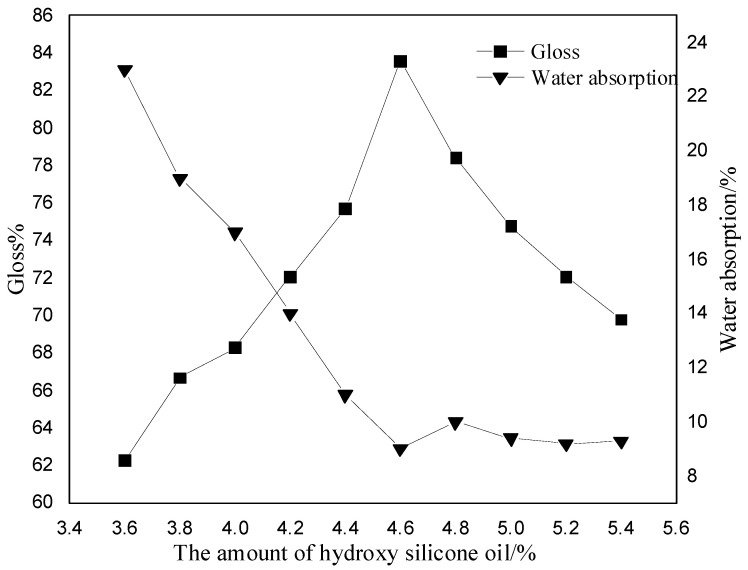
Effect of the amount of hydroxy silicone oil on gloss and water absorption.

**Figure 7:**
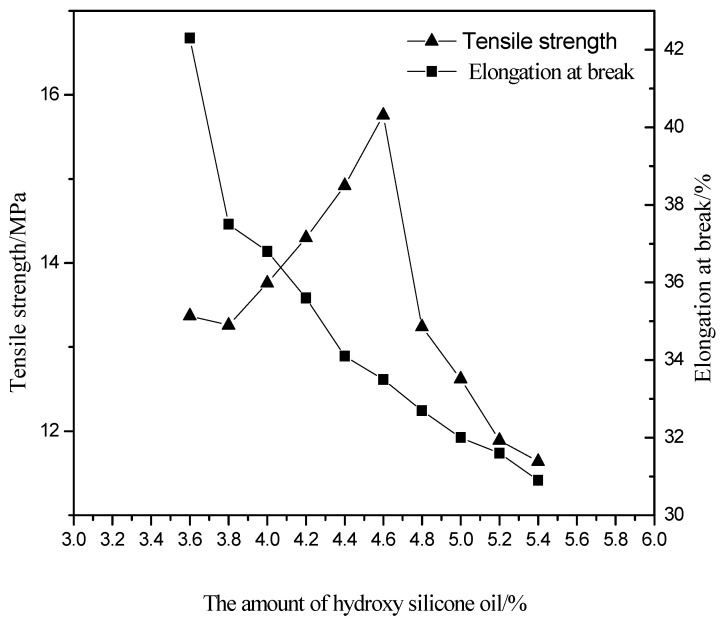
Effect of the amount of hydroxy silicone oil on tensile strength and elongation at break.

As can be seen from Figure 6, with the increase in the amount of hydroxy silicone oil, the glossiness first increases and then decreases, with the highest glossiness at 4.6%; the water absorption rate decreases with the increase in the amount of hydroxy silicone oil and then remains stable. The reason for this phenomenon is that hydroxyl silicone oil has a large number of hydroxyl groups, which can be copolymerized with isocyanate roots to obtain a Si-O-C bond. The Si-O-C bond is introduced into the polymer macromolecule chain as soft segments, so that the structure of the polymer macromolecule changes, and the flexibility of the molecular surface increases, the surface becomes smooth and the glossiness increases. The amount of hydroxysilicone oil continues to increase and the Si-O-C content increases, but due to the poor compatibility of the silicone bond with the isocyanate root, micro-phase separation occurs, results in a reduction in glossiness. With the addition of hydroxyl silicone oil, the silicon atom adheres to the membrane surface, increasing the hydrophobicity of the membrane, slowing the rate of water molecules entering the interior of the membrane, and making it difficult for the resin to bond with the water molecules, thus decreasing the water absorption rate. With the increase of dosage, the number of silicon atom on the membrane surface is gradually saturated, and thus the water absorption rate remains basically stable. Figure 7 shows that as the dosage of hydroxysilicone oil increases, the tensile strength increases and then decreases, and the elongation at break decreases. The reason for this is that the addition of hydroxysilicone oil will cause micro-phase separation within the polyurethane molecule, and the increase in amount increases the degree of micro-phase separation, increasing the tensile strength of the light-cured film and decreasing the elongation at break. When the amount of hydroxysilicone oil continues to increase to 4.6%, the degree of microphase separation is too high, which will lead to an increase in defective structures within the molecule and a decrease in mechanical properties. Table 1 shows that as the amount of hydroxysilicone oil increases, the hardness of the light-cured film first increases and then begins to decrease when the amount exceeds 4.8%. The adhesive level increases with the increase of hydroxysilicone oil. The reason for this is that when hydroxyl silicone oil is added, the reaction results in Si-O-C bonding, which strengthens the intermolecular forces and therefore increases the hardness of the light-cured film and gradually improves the adhesive level. Therefore, the optimum amount of hydroxy silicone oil is 4.6%.

### 3.6. Effect of the Amount of Dimethylol Propionic Acid (DMPA) on Product Properties

**Figure 8:**
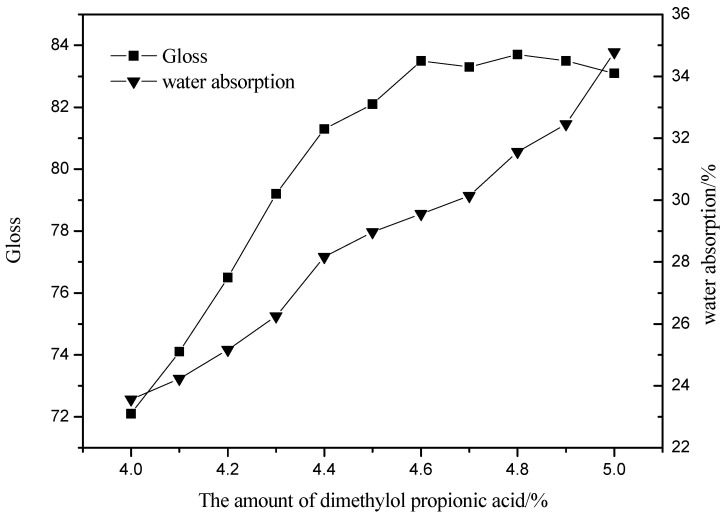
Effect of dimethylol propionic acid amount of gloss and water absorption.

As can be seen from Figure 8, with the amount of DMPA increasing, the glossiness of the light-cured film increases first, and the glossiness basically remains stable when the amount exceeds 4.6%. The water absorption rate increases with the increase of the amount of DMPA. As can be seen from Table 2, the hardness of the light-cured film increases as the amount of DMPA increases. When the amount exceeded 4.6%, the hardness remained constant. The adhesion of the light-cured film increased with the increase of the amount of DMPA. The reason for this is that DMPA contains -OH, which can react with isocyanate in a copolymerization reaction, increasing the cross-linking of molecules, the smoothness of the molecular surface, the glossiness and hardness gradually increase. The number of bonds on the molecular chain that can interact with each other increases accordingly, making the adhesive force increase. When the amount of DMPA exceeds 4.6%, the degree of intermolecular cross-linking remains basically unchanged, so the hardness remains basically unchanged and the glossiness and adhesion remain stable, but the content of hydrophilic groups increases, which is reflected in the increase of water absorption and the deterioration of water resistance of the light-cured film. When the amount of DMPA is too much, the water absorption rate of the molecule is too large, and it is easy to cause the swelling or even dissolution of the molecule.

In order to obtain the product with good performance, the optimum DMPA amount is 4.6%.

### 3.7. Effect of Polyethylene Glycol-1000 Amount on Product Properties

**Figure 9:**
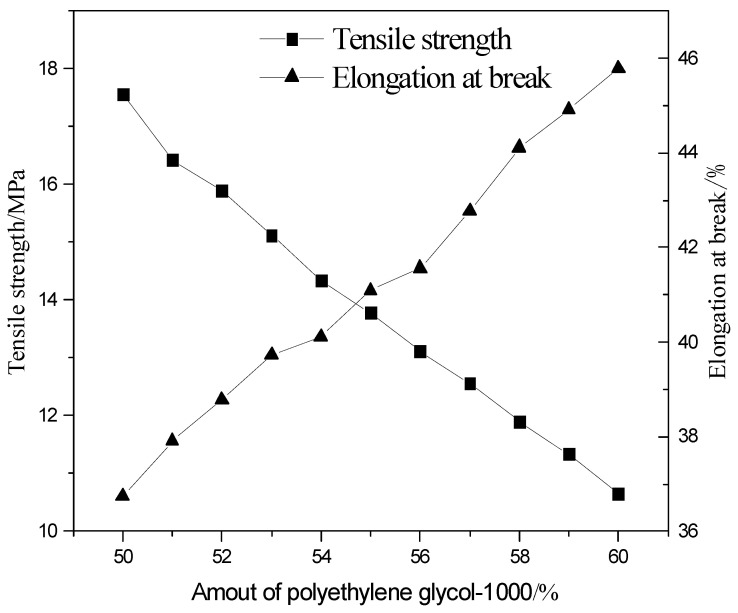
Effect of polyethylene glycol-1000 the amount of tensile strength and elongation at break.

As can be seen in Figure 9, the tensile strength of the light-cured film decreases as the amount of polyethylene glycol-1000 increases, while the elongation at break increases. Table 3 shows that the adhesive grade gradually decreases with the increase in the amount of polyethylene glycol-1000. The hardness and glossiness level then decreases. This is due to the fact that with the increase in the amount of polyethylene glycol-1000, the content of rigid groups (mainly urethane) in the hard segment of the molecular chain decreases. Meanwhile, the content of flexible groups (mainly ether bond) in the soft segment increases, and the interaction force between molecules decreases, which increases the flexibility of the cured film, decreases the tensile strength and reduces the adhesion, and at the same time, because of the increase in the flexible links, the flexibility of the molecular chain is good, which causes elongation at break. The elongation at break rises. The increase in amount leads to a decrease in the cross-linking of the molecules, which further reduces the intermolecular interaction forces and further the hardness. At the same time, the molecular structure of the molecular chain is constantly changing and the proportion of soft segments in the molecular chain is increasing, which reduces the surface refractive properties of the molecular chain and leads to a decrease in glossiness.

According to the above analysis, the optimal amount of polyethylene glycol-1000 in this experiment is 50%.

### 3.8. Effect of Hydroxyl-Terminated Hyperbranched Polymer Dosage on Product Performance

**Figure 10:**
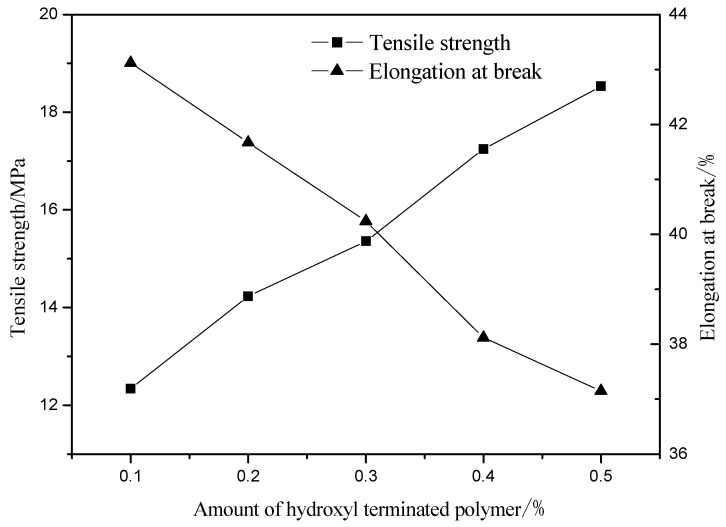
Effect of the amount of hydroxyl terminated polymer on elongation at break strength grade.

With the increase of the amount of hydroxyl-terminated hyperbranched polymer, the hardness increases and the water absorption of the cured film is decreased (Table 4). That is because there is a large amount of -OH that can continuously react with -NCO, the hard segment is continuously introduced, the rigid group content increases, and the intermolecular force is enhanced. At the same time, the hyperbranched polymer has a large number of terminal hydroxyl groups which increase the degree of cross-linking during the reaction, and the force between molecules is further enhanced. Therefore, the hardness of cured film increases. The degree of cross-linking of the molecules increases during the reaction, making it difficult for water molecules to pass through the membrane to enter the interior of the molecules, which lead to reduce the water absorption rate. As shown in Figure 10, with the increase in the amount of hydroxyl-terminated hyperbranched polymer, the breaking strength of the cured film continues to increase, and the elongation at break continues to decrease. The reason for this is that the terminal hydroxyl groups of the hyperbranched polymer continuously react with isocyanate to form carbamate, which increases the length of the molecular chain and the number of rigid groups, the breaking strength and decreases elongation at break of the coating film. Therefore, the most suitable quantity of hydroxyl-terminated hyperbranched polymer is 0.5%.

### 3.9. Effect of Reaction Temperature and Catalyst on -NCO Conversion Rate

**Figure 11:**
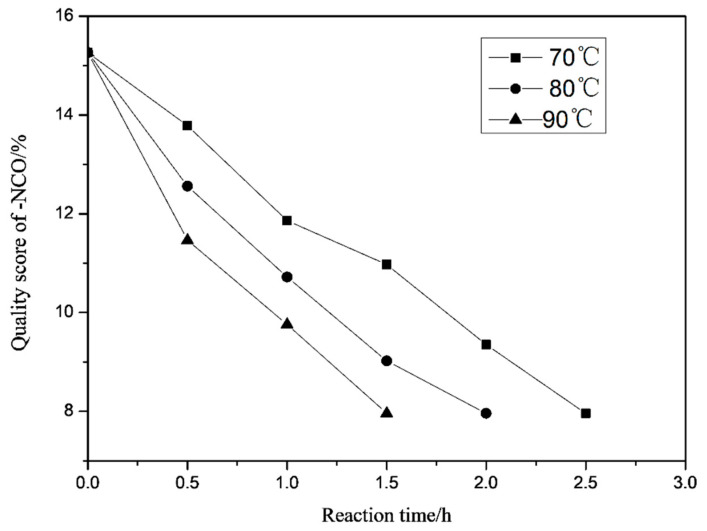
Effect of reaction temperature on -NCO conversion rate.

As can be seen in Figure 11, when the reaction temperature is 70 °C, 80 °C and 90 °C, the reaction time when the end of the reaction respectively is reached is 2.5 h, 2 h and 1.5 h. When the reaction temperature is 70 °C, the reaction takes 2.5 h to reach the end of the reaction, although the reaction process is easier to control, the reaction time is too long and the efficiency is low; when the reaction temperature is 90 °C, although the reaction rate is faster, a lot of heat is produced during the reaction; it is difficult to control the reaction process, and side effects easily occur. When the reaction temperature is 80 °C, it takes 2 h to reach the end of the reaction. The reaction rate is moderate, and the reaction process is relatively easy to control.

**Figure 12:**
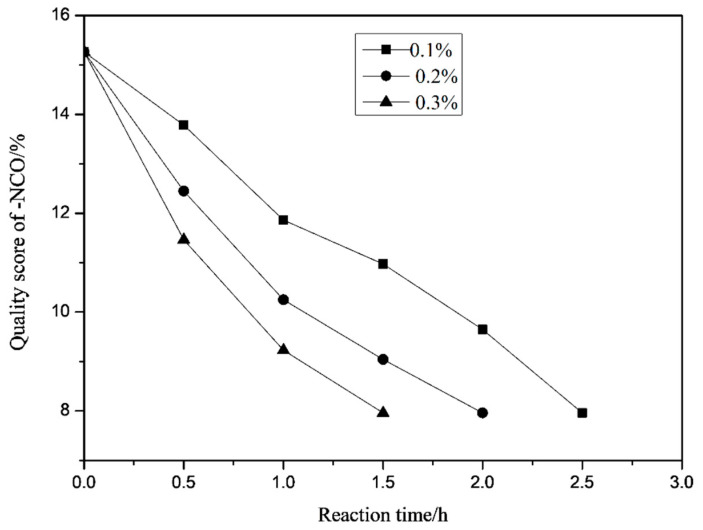
Effect of catalyst dosage on -NCO conversion rate.

As can be seen in Figure 12, with the increase of the amount of the catalyst DBTDL, the reaction time gradually decreases, and the reaction rate increases. However, when the amount of the catalyst is too much, the reaction rate is too fast, gel is easy to occur. Also, the reaction exotherms too fast, which affects the control of the temperature and thus affects the formation of products. When the amount of DBTDL increases, it will also reduce the storage stability and chemical properties of the polymer. Considering comprehensively, the optimal amount of catalyst is 0.2%.

### 3.10. Properties of UV Cured Films after Coating

The experiment used the best conditions which were explored when the amount of hydroxy silicone oil was 4.6%, the amount of dimethylol propionic acid was 4.6%, the amount of polyethylene glycol-1000 was 50%, and the amount of hyperbranched prepolymer was 0.5%, the reaction temperature of silicone modified was 80 °C, the reaction time was 2 h, and the amount of catalyst was 0.2% to prepare hyperbranched silicone waterborne polyurethane acrylate. It verified the comprehensive properties of the coating film, such as the glossiness, hardness, adhesion, abrasion resistance, water absorption, tensile strength, and elongation at break.

As shown in Table 5, the light-cured film after coating has excellent properties and good adhesion with white cardboard, and it has good glossiness, hardness, tensile strength, elongation at break, water absorption and abrasion resistance compared with the cured film of waterborne polyurethane acrylate oligomer that has not been modified by hyperbranched silicon.

## 4. Conclusions

This experiment synthesized hyperbranched silicon modified waterborne polyurethane acrylate which was modified with hydroxyl-terminated hyperbranched polymer, hydroxy silicone oil, and dimethylol propionic acid, and the end-capped synthesis was carried out with acrylate-β-hydroxyethyl. Moreover, we studied the optimal reaction conditions and drew the following conclusions:

1. Hydroxy silicone oil as a modifier mainly affects the hardness, adhesion, glossiness, tensile strength, elongation at break, and water absorption of the polyurethane light-cured film. When the mass fraction of hydroxy silicone oil is 4.6%, the hardness, adhesion and glossiness of the light-cured film are better, the tensile strength and elongation at break of the film are good, the water absorption rate is low, and it has a certain degree of water resistance.
2. As a water-based monomer, dimethylolpropionic acid also has a certain cross-linking effect, which mainly affects the glossiness, water absorption, and hardness of the light-cured film, and has a certain impact on the adhesion. When the mass fraction of dimethylolpropionic acid is 4.6%, the light-cured film has higher glossiness, hardness and adhesion. At the same time, it has certain water resistance under the premise of certain water absorption.
3. As a soft segment material, polyethylene glycol-1000 mainly affects the glossiness, tensile strength and elongation at break, adhesion and hardness of the light-cured film. When the mass fraction of polyethylene glycol increases, the number of soft segments increases, and the number of rigid groups in the molecule decreases, resulting in a decrease in tensile strength, an increase in elongation at break, and a decrease in adhesion and hardness. When the amount of polyethylene glycol-1000 is 50%, the tensile strength, hardness, and adhesion of the film are the highest, while the elongation at break decreases.
4. The hyperbranched polymer with terminal hydroxyl as a modified monomer mainly affects the hardness, water absorption, tensile strength and elongation at break of the light-cured film. When the hyperbranched polymer with terminal hydroxyl mass fraction is 0.3%, the hardness is higher, and the water absorption, tensile strength, and elongation at break are good.

## Figures and Tables

**Table 1:** Effect of the amount of hydroxy silicone oil on hardness and on adhesion.

Amount of Hydroxy Silicone Oil/%	Tensile Strength	Elongation at Break
3.6	3B	Level 4
3.8	2B	Level 4
4.0	2B	Level 3
4.2	HB	Level 2
4.4	2H	Level 1
4.6	3H	Level 0
4.8	3H	Level 0
5.0	2H	Level 0
5.2	HB	Level 0
5.4	2B	Level 0

**Table 2:** Effect of dimethylol propionic acid content on the hardness and adhesion.

Amount of Dimethylol Propionic Acid/%	Tensile Strength	Elongation at Break
4.0	B	Level 3
4.1	B	Level 3
4.2	HB	Level 3
4.3	2H	Level 2
4.4	2H	Level 3
4.5	3H	Level 2
4.6	4H	Level 1
4.7	5H	Level 1
4.8	5H	Level 1
4.9	5H	Level 1
5.0	5H	Level 1

**Table 3:** Effect of amount of polyethylene glycol-1000 on the hardness and adhesion.

Amount of Polyethylene Glycol-1000 the/%	Elongation at Break	Tensile Strength	Gloss
50	Level 1	3H	87
51	Level 1	3H	86
52	Level 1	H	85
53	Level 2	H	85
54	Level 2	HB	83
55	Level 3	HB	82
56	Level 3	B	81
57	Level 3	B	79
58	Level 3	2B	77
59	Level 4	3B	76
60	Level 4	3B	74

**Table 4:** Effect of terminal hydroxyl hyperbranched polymer content on the hardness.

Amount of Hydroxyl-Terminated Hyperbranched Polymer/%	Hardness	Water Absorption (%)
0.1	2B	17.5
0.2	HB	16.2
0.3	HB	15.7
0.4	H	14.8
0.5	2H	13.3

**Table 5:** The performance of light cured coating film.

Specimen	Gloss	Hardness	Adhesion	Abrasion Resistance	Breaking Elongation	Water Absorption	Tensile Strength
Hyperbranched silicon modified waterborne oligomers	98.7	4H	level 1	19.31 MPa	6.73%	9.21%	good
Common waterborne oligomers	87.2	2H	level 2	14.21 Mpa	4.21%	8.23%	good

## Data Availability

Data sharing not applicable.
